# The Week's Work

**Published:** 1911-02-18

**Authors:** 


### The Week's Work.

### BRADFORD'S PROPOSED INFIRMARY.

There appears to be a disposition towards apathy at Bradford in regard to the proposed new Infirmary, and it has been stated that in the short space of a fortnight nearly as much money has been raised at Leeds for the necessary improvements to the Infirmary there as has been collected in Bradford in two years. Public notices exhibited on the site in Duckworth Lane, Bradford, have made it clear that the funds raised must be sufficient to build, equip, and adequately endow the new Bradford Hospital, and yet it is the view in some quarters that donations are being withheld on the ground that the Board of Management will be unable to maintain the new Infirmary when it is built, and that it will be put upon the rates. The local Press has drawn attention to these points in the course of an endeavour to stimulate practical and monetary sympathy, and it has been pointed out that one of the best-known experts in hospital construction has expressed the opinion that the site which has been obtained is a magnificent one, and if Bradford will rise to the occasion there is a great future for the new Bradford Infirmary. We pointed out more than a year ago (November 6, 1909, p. 161) the urgency of the need for rebuilding, and more lately referred to the essentials of the rebuilding scheme (January 28, 1911, p. 539). Those who have interested themselves in the matter cannot but regret this causeless hesitancy on the part of possible contributors. The staple trade of the city is in a flourishing condition, and the prospects for the future are bright. Everyone who can should undoubtedly assist —and that immediately—the scheme, so that Bradford may have an Infirmary which shall at once be efficient and adequate and shall be a striking tribute to the late King Edward.

### THE VOLUNTARY HOSPITAL AND THE PUBLIC ASSISTANCE AUTHORITY.

Under the title "The Function of the Voluntary Hospital in Relation to the Proposed Public Assistance Authority," Mr. J. Courtney Buchanan republishes in pamphlet form the essay from his pen which appeared in the *Hospital Gazette* last year. Mr. Buchanan is Secretary of the Metropolitan Hospital, a well-known member of the Economics School of the London University, and a barrister-at-law—a combination which makes his opinion on one of the most important questions of the day, particularly as he speaks with the authority that comes from a long and earnest study of the subject, especially interesting and worhty of consideration. Taking as the basis of his thoughtfully written pamphlet the paper which he read before a recent meeting of the Hospital Officers' Association, he deals seriatim with seven separate but closely connected items: charitable contributions, in-patients, out-patients, co-operation with the Public Health Service, co-operation with the Poor Law service, classification, and general control. In discussing these items he is always thorough, always moderate, and generally instructive. We are not always in close agreement with him, for instance, to quote only one opinion, with regard to the advisability of establishing a central buying station—but we recognise fully that so able and original a thinker has a right to be precise, and even dogmatic, in his statements. The pamphlet (which can be bought at the bookstalls for a shilling) is worth the attention of everyone who takes an interest, whether active or dilettante, in our hospitals, and especially of those who are striving to preserve the voluntary system. Mr. Buchanan's arguments and his able exposition of the case for voluntaryism should win him many readers from both camps. We hope to return to his work for a more extended review in a future issue.

### THE PAY SYSTEM AT CARDIFF INFIRMARY.

At the last monthly meeting of the Board of Management of the Cardiff Infirmary, held last week, an important recommendation was submitted by the Ways and Means Committee. This was a scheme to provide for a small contributory rate from in-patients and the levying of an even smaller registration fee for out-patients. The Committee recommends that each in-patient be asked to contribute sixpence daily towards provision expenses, and that each out-patient should be charged threepence as a registration fee. Colonel Bruce Vaughan, in explaining the scheme, said its success would mean an increase of £700 at least in the annual income of the institution. None of the patients would be called upon to pay unless they could afford to do so. After some discussion the Board decided to try the scheme, as an experiment, for a year, with the important amendment that it is to be left to the subscriber signing the patient's ticket to state whether or not the patient is in a position to pay the fee or the contribution. It is perhaps impossible to rob the subscriber entirely of the right to admit patients free to a hospital to which he or she subscribes. So long as the inducements held out to subscribers of most voluntary institutions remain what they are, it is equally impossible for the most progressive of hospital committees to deal adequately, thoroughly, and effectively with the question of hospital abuse, especially in the out-patient department. We are therefore not surprised to note that a body of subscribers—the miners of Merthyr Vale, to be precise—has already protested against the innovation, and it is likely that other subscribers will follow suit.

### WHAT IT MEANS.

For all that, we congratulate the management of the Infirmary on its progressiveness that has made it adopt the scheme. We shall watch the working of the new regulations with interest, though we do not anticipate that the innovation will prove a failure, for we are convinced that subscribers, once they have really perceived what the suggestions mean and what they have as their object, will agree heartily with the Committee's recommendations. As a mere means of raising money the new rules are not to be approved of: there are other and less revolutionary methods of raising £700 yearly for the benefit of the Infirmary. But when it is remembered that they are designed to raise the status of the institution, to make Cardiff Infirmary one of the best types of voluntary institutions, the type in which the patient realises that he, too, has an obligation which must be responded to so far as his means permit him to do so, it will be seen that their importance is very great and that they may be potent for good. The contributory basis appears to us to be excellent for such a scheme as proposed, and the amount suggested to be reasonable. Perhaps the in-patient contributory fee might well be lowered to threepence in cases where the levy of a sixpence daily, small as that sum is, seems too much for poorer patients. By at once classing all inmates who cannot afford the sixpenny levy as "paupers" an injustice is likely to be done to many self-respecting sick people who would be only too glad to give a small contribution daily (from a penny upwards) towards their keep. By accepting such a small contribution, however insignificant it may seem to hospital authorities, who forget that the contributory scheme is not so much designed to bring revenue to the hospital as to influence patients and the public alike, a hospital will show that it recognises the willingness of this class of patient to help, and, what is much more, his eagerness to maintain a position of self-dependence and self-respect. The out-patient registration-fee might with advantage be similarly graded, from a penny upwards.

### THE WEEK'S DRUG MARKET.

The state of the drug market continues very interesting and the advancing tendency of prices is maintained. Notwithstanding the large increase in the value of menthol which has recently taken place, the price is again substantially higher; the demand, however, is quiet, as buyers are reluctant to procure more than they require for immediate use at the present rates. Oil of peppermint also continues to advance in price. A large business has been done in refined camphor, which is again dearer. The expected advance in the prices of potassium, ammonium, and sodium bromides has now been announced by the makers. The high value of carbolic acid is maintained and the large demand for the Far East continues. Buyers of cod-liver oil are inclined to delay their orders until more definite news concerning the fishing is received from Norway, but in the meantime the advanced price is maintained. Turpentine is dearer. The tendency of prices of opium continues to be in an upward direction and higher quotations for morphine and codeine are not expected. Calumba root is fetching advance in value. Gamboge is slightly cheaper. It is generally considered that the price of glycerine has reached its highest limit and that no alteration is to be expected at present.

